# Using normalization process theory to evaluate the implementation of a hybrid psychosocial prevention intervention in mental health care – a qualitative interview study

**DOI:** 10.1186/s12913-026-14631-x

**Published:** 2026-05-13

**Authors:** Jan Gehrmann, Johannes Stephan, Jana Dehner, Ananda Stullich, Matthias Richter

**Affiliations:** 1 Chair of Social Determinants of Health, TUM School of Medicine and Health, Department Health and Sport Sciences, Technical University of Munich, Munich, Germany; 2 Institute of General Practice and Health Services Research, TUM School of Medicine and Health, Department Clinical Medicine, Technical University of Munich, Munich, Germany

**Keywords:** Mental health, Digital health, Hybrid intervention, Prevention, Implementation, Normalization process theory, Medical apps

## Abstract

**Background:**

The increase in mental health disorders underscores the importance of preventive interventions and strategies. The approach of hybrid interventions integrating analog and common therapeutic approaches with digital health applications offers promising solutions but faces challenges in routine implementation. This study examines the implementation of the new hybrid intervention ‘RV Fit Mental Health’ within the German health care system, using Normalization Process Theory (NPT) as the guiding theoretical framework.

**Methods:**

A qualitative study was conducted with nine experts involved in the development and implementation of the intervention, including professionals from the therapeutic and administrative domains of the implementation. Data were collected through semi-structured interviews and analyzed using qualitative content analysis, following the NPT framework and the NPT coding manual.

**Results:**

The findings demonstrate that while the shift towards preventive care within the intervention required substantial organizational and normative adaptations, the intervention was largely perceived as a valuable and integrative addition in established care. Facilitating factors included stakeholder collaboration, a shared understanding of and goals for preventive care, and flexible, open organizational structures. Barriers and challenges were mainly linked to time and resource constraints, technical integration of the digital component, and the redefinition of professional roles. The implementation of the intervention reshaped therapeutic routines, fostered interdisciplinary collaboration, and was increasingly normalized within the specific conditions of a model project, both within clinical and administrative workflows and across organizational boundaries.

**Conclusions:**

The implementation of the hybrid intervention RV Fit Mental Health was largely successful. The evaluation of the implementation of (hybrid) interventions benefits from a theory-driven approach. NPT provided valuable insights into how such interventions are embedded in existing systems and structures and how preventive and hybrid approaches can be sustainably integrated. The results offer both theoretical and practical implications for the design, implementation, and evaluation of similar interventions in diverse healthcare settings.

**Trial Registration:**

DRKS-ID: DRKS00033080 (Date of registration: 07 December 2023)

**Supplementary information:**

The online version contains supplementary material available at 10.1186/s12913-026-14631-x.

## Background

As mental health disorders are steadily increasing in recent years and are contributing to long-term social and economic consequences, preventive policy strategies and interventions are becoming more important [1], [2–4]. Digital health applications have been shown to be a promising approach to mental health prevention [5, 6]. Especially the flexibility, the integration into everyday life, and accessibility, as well as the access, offer great potential for digital health, particularly for mHealth applications such as smartphone- or tablet-based tools that support health monitoring, psychoeducation, and behavioral interventions outside of clinical settings [1, 7–9]. Recent developments in this field also highlight the potential of hybrid models that combine digital applications with traditional therapeutic methods to enhance accessibility, engagement, and long-term sustainability of preventive care [6, 10–12]. Similarly, studies focusing on mHealth applications (mHealth apps) in outpatient psychosocial care emphasize their potential to improve service delivery and patient outcomes by offering flexible, self-directed support [12, 13]. Despite the promise of these interventions, challenges remain in effectively implementing and scaling digital health applications in routine care settings and their potential use [14–17]. This underscores the need for further exploration of the factors that influence the successful integration of digital applications in (preventive) care, particularly in terms of acceptance of health care professionals and administrative personnel, system and organizational integration, and long-term practical use, as analyzing the context of new interventions is crucial for implementation research [18–23].

In the PE^3^PP project (Germany Acronym for “Development, Piloting and Evaluation of a psychosocial prevention intervention”), the new hybrid prevention intervention RV Fit Mental Health was developed and implemented [24]. Prevention care is conceptualized as an overarching approach to structured preventive interventions. The present intervention is designed as a secondary preventive intervention targeting individuals with early or subclinical psychological, psychosomatic, and psychosocial burden in combination with work-related participation disorders, whose conditions are related to or exacerbated by work-related demands. Eligible participants typically include individuals with affective disorders, anxiety disorders, adjustment disorders, somatoform disorders, or burnout. To operationalize this approach, Caplan’s stage-based model guides the intervention content by aligning preventive strategies with symptom severity, while Gordon’s risk-based framework structures access pathways and identifies individuals with varying levels of vulnerability.

Accordingly, the intervention integrates three access pathways: an indicated pathway including individuals with an ICD-coded mental health diagnosis and a denied rehabilitation request, a selective pathway including individuals identified through prolonged sick leave due to mental health conditions, and a universal pathway allowing voluntary participation via public outreach. Participants entering through selective and universal pathways undergo standardized screening to ensure alignment with the secondary prevention focus, while individuals requiring primary or tertiary interventions are referred to appropriate services. Within the intervention, individualized prevention goals are defined based on a structured needs assessment and addressed through a combination of core modules and tailored components. By integrating risk-based access with stage-based intervention design, the program enables a structured and needs-based approach to secondary prevention. Further details on the underlying classification approach are provided elsewhere [25].

**Fig. 1:**
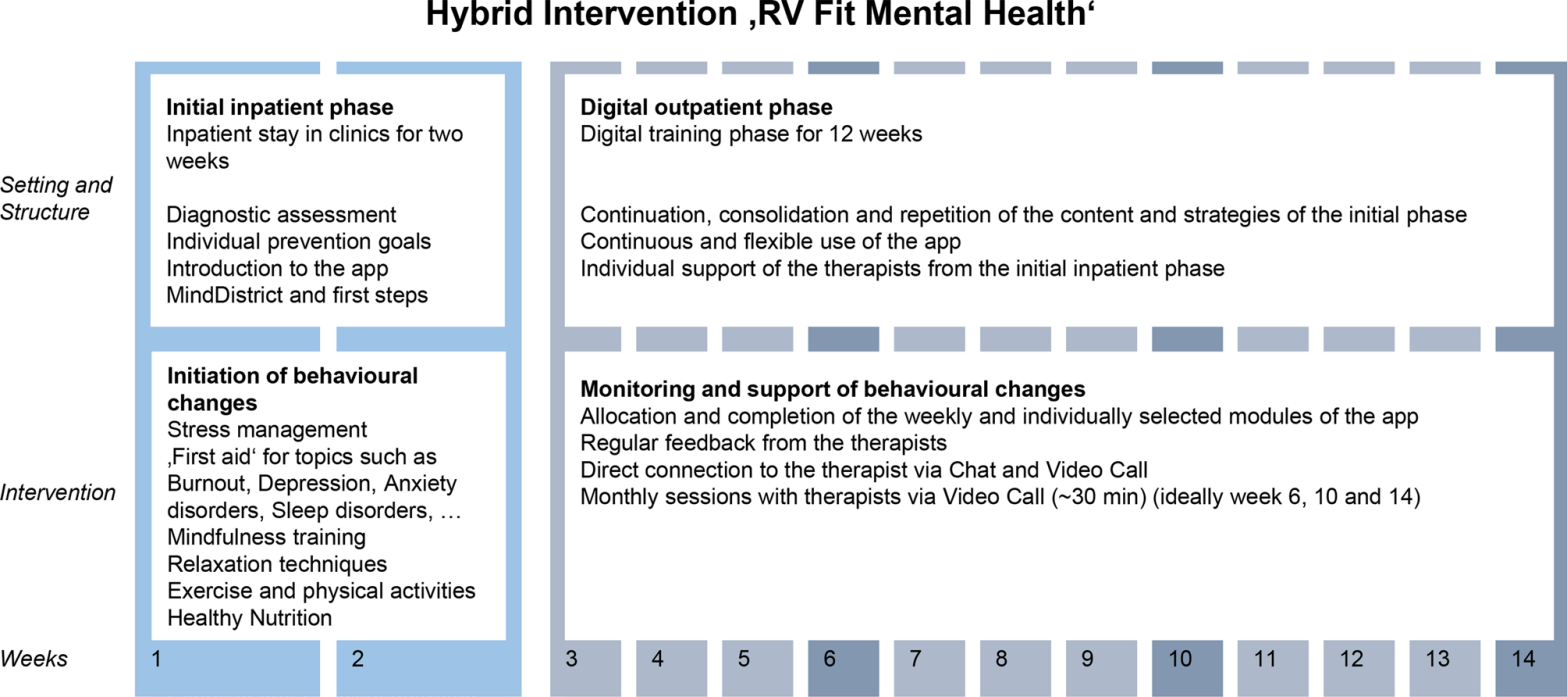
Overview of the study intervention

The intervention consists of a two-week inpatient phase followed by a 12-week outpatient digital training phase, supported by a medical app and therapeutic support via chat, telephone, or video call. Figure 1 provides an overview of the intervention components and its phases. As the intervention is completely new and initially implemented within a model project, an in-depth perspective on the implementation of the hybrid approach is crucial to ensure its integration into complex institutional structures, acceptance among health care professionals, administrative staff, and potential users [21, 22]. Despite the growing push for digital transformation in health care services, there remains a significant research gap in understanding how such interventions can be successfully adopted, scaled, and sustained within varying contexts [17, 20, 26–30].

Therefore, a theoretical implementation framework tailored to the intervention setting and the specifics of the newly developed intervention is beneficial, also offering an in-depth understanding of the processes and the framework conditions surrounding its implementation [27, 31]. To evaluate this implementation, we applied Normalization Process Theory (NPT) as the guiding theoretical framework. It offers a theoretical framework for evaluating the implementation of (innovative) complex interventions and has shown its potential also in mental health interventions [32–35].

This paper aims to evaluate the implementation of a hybrid psychosocial prevention program. By examining the practical and theoretical aspects of intervention implementation, this study contributes to the ongoing discourse on how to optimize the use of digital health technologies for prevention.

Therefore, the research questions of this study are:

- *How is the new hybrid prevention intervention RV Fit Mental Health implemented and normalized, as evaluated using NPT as a theoretical framework?*
- *Which facilitating factors and barriers shape the implementation process from the perspective of professionals involved in the development and delivery of the intervention, and how do these influence its normalization within clinical and administrative workflows?*

## Methods

### Study design and setting

The PE^3^ PP project is funded through the federal program ‘Innovative Ways to Participate in Working Life – rehapro’ of the Federal Ministry of Labor and Social Affairs, Germany. The project runs from October 2021 to October 2026. The German Pension Insurance Central Germany leads the project (German: ‘Deutsche Rentenversicherung Mitteldeutschland’; DRV), further project partner are the health insurance company ‘Allgemeine Ortskrankenkasse PLUS’ (AOK PLUS) as well as two rehabilitation clinics in central Germany (MEDIAN Klinik Bad Gottleuba and SRH Medinet Burgenlandklinik) who developed and are currently conducting the intervention. The intervention is implemented in an inpatient rehabilitation clinic setting, with the subsequent digital training phase conducted remotely via the MindDistrict app. The Chair of Social Determinants of Health at the TUM School of Medicine and Health at the Technical University of Munich (TUM) is responsible for the scientific accompaniment of the project and evaluation of the intervention. The overall study is a prospective multicentric study, with the two named clinics conducting the intervention from 2023 to 2026.

The intervention focuses on the psychological, psychosomatic, and psychosocial aspects of work-related participation disorders and is implemented in cohorts. The intervention explicitly targets mental health problems that are related to or exacerbated by the work environment, including affective disorders, phobic and other anxiety disorders, adjustment disorders, somatoform disorders, and burnout. In terms of prevention classification, RV Fit Mental Health constitutes secondary prevention within Caplan’s framework and addresses selective and indicated prevention populations within Gordon’s risk-based framework [25]. The app provides various intervention content and modules during the digital training phase, including exercises, instructions, and training materials in text, video, and audio formats, and ensures a digital bridge between participants and clinic staff. As a complex intervention, it comprises components integrated across both the inpatient and digital training phases. Figure. 1 gives an overview of the intervention. Further details of the intervention and the study background are covered in the study protocol and in Supplementary Material 1 [24].

The theoretical framework of this study is the NPT [34, 35]. NPT aims at behavioral and organizational (structural) aspects of new practices and how these will be successfully adopted and became routinized and thus normalized, and in that sense, NPT focuses on interactional (individual and communal) as well as structural dynamics in the processes of innovations [34, 36]. It contributes to a holistic and detailed evaluation of implementation processes and, therefore, to guidance on the practical and administrative aspects of implementation.

For this qualitative study evaluating the intervention, experts involved in the intervention’s administrative and therapeutic development and implementation were interviewed [37, 38]. The study is reported in accordance with the COnsolidated criteria for REporting Qualitative research (COREQ) (see Supplementary Material 2) as well as the NPT framework [39–41].

### Sample and recruitment

To cover a variety of perspectives and experiences in the implementation of the new intervention, purposive sampling was applied [42]. Relevant participants were identified by the research team. Inclusion criteria were: (1) direct involvement in the development and/or implementation of the intervention, either in an administrative, organizational, or therapeutic capacity; (2) sufficient German language skills; (3) informed consent. Exclusion criteria were: (1) no direct involvement in the project; (2) a role already fully covered by another interview participant. These criteria ensured that participants held first-hand knowledge of the implementation process from a variety of professional perspectives. Interview participants were contacted via phone or email. Prior to data collection, the study information and the informed consent form were sent via email, along with confirmation of the interview date. In total, 13 individuals met the inclusion criteria and were identified for recruitment. Three could not be interviewed as they had temporarily or permanently withdrawn from the project, and one person did not participate as their role was already fully covered by another interview participant. Overall, nine interviews were conducted. The decision to conclude recruitment after nine interviews was based on the purposive sampling strategy and the well-defined expert population. Given that 13 individuals met the inclusion criteria, of whom nine were ultimately interviewed, the sample covered a broad range of relevant perspectives across all project partners and professional domains (therapeutic, administrative, organizational). The research team felt that thematic saturation had been reached, as no substantially new implementation-relevant themes emerged across the interviews [43]. Recruitment would have been continued if additional relevant perspectives had been identified.

### Data collection

Interviews followed a semi-structured interview guide [44]. The topics covered in the interview guide are based on the NPT’s constructs. To ensure that all relevant themes are covered, the guide was initially developed according to Helfferichs´ four-step procedure: collecting, reviewing, sorting, and summarizing [45]. The guide was discussed iteratively within the research team for structure, clarity, and congruence with the NPT constructs and the study’s research questions. The interview guide is available in the Additional Files (see Supplementary Material 3). Interviews were conducted by JG and JS by video call or phone as requested. Interviews lasted on average 47 minutes. Interviews were audio recorded and transcribed verbatim by an external agency, using transcription rules according to Dresing and Pehl [46].

### Data analysis

**Fig. 2:**
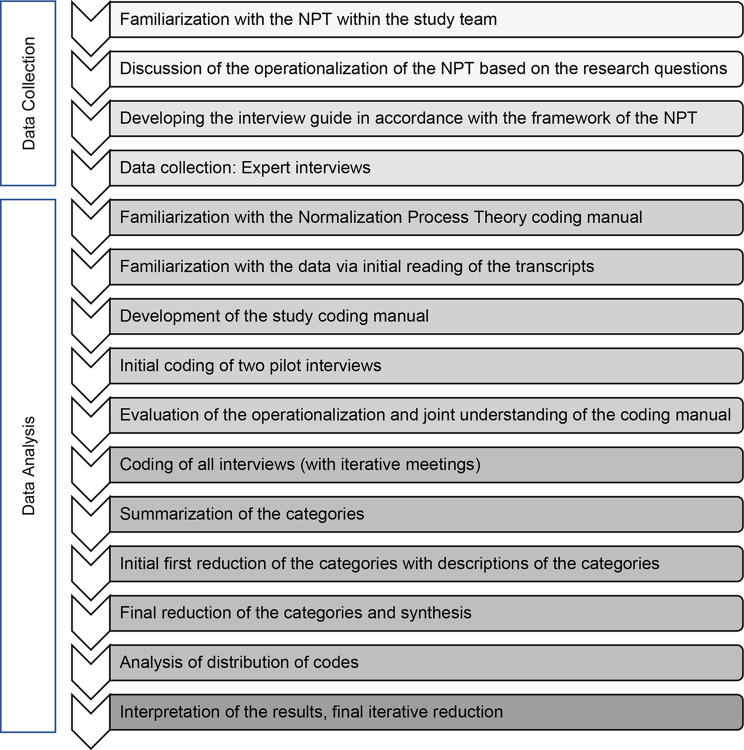
Flow-Diagram of the data collection and analysis

The qualitative data management and analysis software MAXQDA 2022 was used for analysis [47, 48]. Figure 2 shows an overview of the steps of data collection and data analysis.

Before data analysis, we became familiar with the data by reading the transcripts. Data were analyzed using content analysis, and by applying the NPT framework [39, 49, 50]. The NPT coding manual by May et al. was used as theoretical framework [39]. The coding tree was developed deductively based on the coding manual, allowing for the operationalization of the theory and its domains and constructs in a stable and consistent way, aligned with the research questions, study design, and setting.

The coding was done using the pre-determined deductive category system. Two pilot interviews were coded to critically evaluate and foster the understanding of the category system and coding manual, and to ensure no important themes were excluded. We critically assessed the suitability of the NPT coding manual for understanding the phenomena related to implementation processes, explaining the underlying mechanisms, and framing the implementation as a dynamic process. This was vital to ensure the analysis remained flexible, allowing the possibility that the coding framework might be considered either unsuitable for categorizing the data or validated as fit for purpose. A detailed appraisal and operationalization of the NPT-coding manual for our research questions are presented in Table 2.

Subsequently, all interviews were coded by three researchers (JG, JS, and JD) to achieve reliability and intersubjective accountability, followed by reflection within the team on the coding process [51]. Categories were summarized, including anchor examples in each category (with ongoing consultation between a minimum of two authors and close reference to the coding manual). In the first reduction, descriptions of the categories were created, with iterative review by two researchers. In cases of disagreement or discrepancy, a third author was consulted. The synthesis was carried out by JG, leading to the second reduction, which involved refining the category descriptions and the corresponding results. The final reduction was again reviewed by two researchers.

**Fig. 3:**
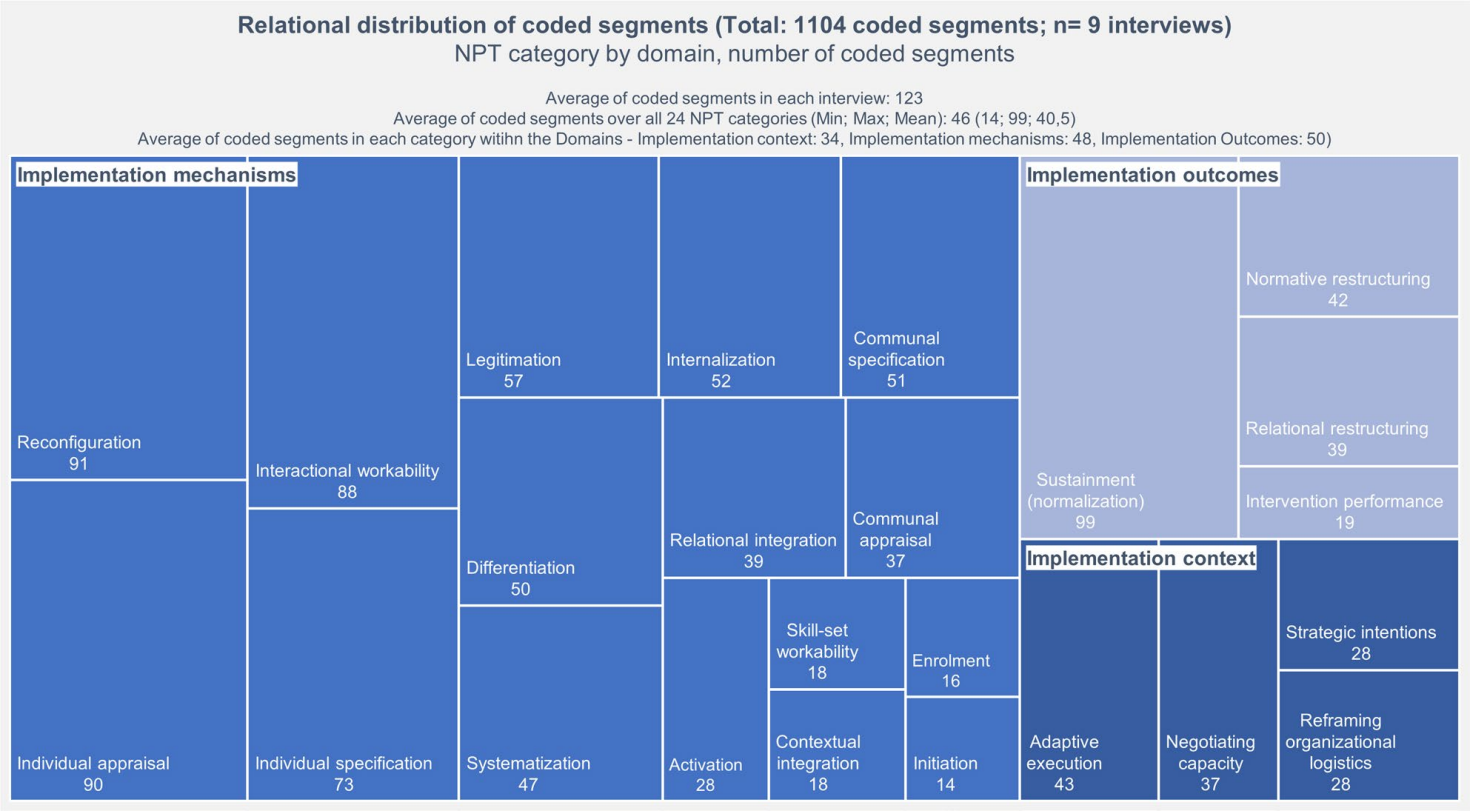
Relational distribution of coded segments within the NPT domains and constructs

The distribution of codes across the NPT domains was analyzed descriptively and is presented in the results section (see Fig. 3).

The overall analysis process, including the study design, was also reviewed within the team (JG, JS, AS, JD, and MR) and the working group for Qualitative Research at the Chair of Social Determinants of Health to ensure rigor and integrity [43, 51]. A minor proportion of data fell outside our coding framework. These data either (1) were not related to the scope of the NPT or (2) focused on the evaluation and optimization of the intervention rather than the nexus of implementation.

Quotes from the data are shown in the results section and are indicated with a pseudonym, the profession (Abbreviations; Physicians and Therapist: “PhTH”; Administration: “Admin”) and the position within the transcript. For visualization of the data and the results, Excel, PowerPoint, and MAXQDA were used [48, 52, 53]. DeepL, Grammarly, and OpenAI were used to support the translation and comprehensibility of the results presented [54–56]. All translations were carefully checked and revised several times by the authors.

## Results

### Sample characteristics

Table 1 presents the socio-demographic characteristics of the participants.

**Table 1:** Socio-demographic details of the participants

Profession	Value (n = 9)
Physicians or Therapists in the clinics (Background in Medicine or Psychology)	5
Administrative staff of DRV and AOK (Background in Healthcare, Administration, Social work, Research or Medicine)	4
**Overall work experience**	
0–9 years	2
10–19 years	1
20–29 years	3
Over 30 years	3
**Work experience in the field of psychosomatic and mental health care**	
0–9 years	5
10–19 years	4
Over 20 years	0
**Age group (in years)**	
30–39	2
40–49	2
50–59	4
n.a.	1
**Gender**	
Female	4
Male	5

### Main results following NPT

Results are presented following the Domains of the NPT: Implementation Context, Implementation Mechanisms, and Implementation Outcomes, each further divided into constructs as outlined in the manual (see Table 2). Table 3 summarizes the key findings across all NPT domains and constructs. Detailed results for each domain are provided in Supplementary Material 4, which contains detailed tables for all constructs and subconstructs within the NPT Domains.

**Table 2:** Overview of the NPT domains, constructs, and their operationalization for this study (following May et al. [39])

Domain	NPT Construct	Objective
Implementation context	Strategic intentions	*How is the implementation influenced by the organizational and structural environment?*
	Adaptive execution	*How does the context affect the ways in which the users can find and enact workarounds that make the intervention and its components workable in practice?*
	Negotiating capacity	*How does the context affect the extent to which the intervention can fit or be integrated into existing ways of working and processes?*
	Reframing organizational logistics	*How do the existing social-structural and social-cognitive resources affect the implementation environment?*
Implementation mechanisms	Coherence building	*How do people individually and collectively make sense of the new intervention and its components to put them into practice?*
	Cognitive participation	*How do people engage and collaborate to conduct and sustain the intervention?*
	Collective action	*How do people work together to enact the intervention and integrate it into the organizations?*
	Reflexive monitoring	*How do people individually and collectively appraise the intervention?*
Implementation Outcomes	Intervention performance	*What practices and processes have changed as the result of the intervention and their components through the operationalization and implementation of the interventions across settings?*
	Relational restructuring	*How has the intervention and their components changed the ways people are organized and related to each other?*
	Normative restructuring	*How has the intervention and their components changed the norms, rules and resources that govern action?*
	Sustainment (normalization)	*How has the intervention and their components become incorporated in (everyday) practice?*

**Table 3:** Summary of key findings following the NPT domains and constructs

Construct	Key findings	Exemplary quote
**Domain: Implementation Context**		
Strategic intentions	• The intervention represented a conceptual shift from rehabilitation to prevention, requiring new approaches to planning and delivery • The intervention addressed a previously unmet need in the care landscape • Clinics had freedom to design content within regulatory constraints	*“It really fills a gap in the offerings” (Exp3, Admin, 55)*
Adaptive execution	• Integration into existing workflows required selective adjustments to documentation, communication, and administrative processes • Over time the intervention became embedded in daily operations • Regular inter-organizational feedback loops helped manage challenges	*“We quickly realized that this is not running smoothly, but over time, as we have now noticed, increasingly small problem areas have popped up” (Exp2, PhTH 3).*
Negotiating capacity	• Implementation was challenging given the tight 14-day inpatient timeframe, requiring immediate activation unlike standard rehabilitation • The preventive approach required different referral processes and clearer selection criteria • Despite challenges, administrative integration was largely successful	*“We have the toolbox anyway, right? So, we have all the colleagues on board, all the possibilities” (Exp2, PhTH, 7)*
Reframing organizational logistics	• Prevention gained greater prominence within rehabilitation clinics • Processes stabilized over time and the intervention became a fixed, plannable element • The intervention complemented rather than disrupted existing rehabilitation routines	*“Prevention has come to the forefront, because it wasn’t as present in our clinic before” (Exp2, PhTH, 29)*
**Domain: Implementation Mechanisms**		
Coherence building	• The intervention was widely perceived as distinct from rehabilitation due to its preventive focus, shorter duration, and digital follow-up phase *(Subconstruct: Differentiation)* • Integration required logistical and cultural adaptation, particularly around workflow changes and staff acceptance *(Subconstruct: Communal specification)* • The intervention was perceived as a valuable and timely development despite implementation challenges *(Subconstruct: Individual specification)* • Strong internalization was evident at both clinical and administrative levels, with staff attributing significant societal value to the intervention and describing it as a meaningful addition to their work *(Subconstruct: Internalization)*	*“It’s a heart project because being able to design something with the participants, to see changes, and to co-create something together is something really valuable” (Exp7, PhTH, 35)*
Cognitive participation	• Key individuals in leadership positions drove the initiation and promotion of the intervention *(Subconstruct: Initiation)* • Staff enrolment involved structured awareness-raising, training, and role clarification *(Subconstruct: Enrolment)* • The long-term benefits of prevention were increasingly recognized as justifying the implementation effort *(Subconstruct: Legitimation)* • The opportunity to co-create an innovative intervention was a key motivator for sustained staff engagement *(Subconstruct: Activation)*	*“This great opportunity to be able to co-create; also to have some room for maneuver in shaping the measure—this opportunity is really unique” (Exp7, PhTh, 43)*
Collective action	• Despite workflow adjustments, task allocation relied on existing skills adapted to the new context *(Subconstruct: Skill-set workability)* • Trust in each other’s expertise and a shared understanding of the program’s procedures facilitated successful implementation *(Subconstruct: Relational integration)* • A new dynamic emerged in the digital phase, with therapists shifting toward supportive and coaching roles *(Subconstruct: Interactional workability)* • Rehabilitation clinics proved well-suited to host prevention efforts given their existing infrastructure *(Subconstruct: Contextual integration)*	*“That is why everyone has to know: Who fills out what, and where? These things were all uncharted territory for us, but it has leveled out well” (Exp2, PhTh, 15)*
Reflexive monitoring	• Structured stakeholder communication enabled continuous access to information about the intervention’s effects *(Subconstruct: Systematization)* • Initial communal appraisal was marked by concern over disrupted routines, which gradually shifted to positive perception *(Subconstruct: Communal appraisal)* • The intervention was regarded as immediately beneficial and promising in terms of long-term outcomes *(Subconstruct: Individual appraisal)* • Continuous reconfiguration of the 14-day schedule and digital phase reflected a commitment to iterative improvement *(Subconstruct: Reconfiguration)*	*“Regular feedback from scientific monitoring would be beneficial—are there any necessary changes? Are there critical points that perhaps participants have not mentioned directly?” (Exp7, PhTh, 23)*
**Domain: Implementation Outcomes**		
Intervention performance	• The structured 14-day schedule added clarity and predictability to clinical routines • Technical enhancements and staff training supported efficient implementation • Participant feedback confirmed strong acceptance, with the measure described as well to very well received	*“This measure is well to very well received. And that we also see it as a success for ourselves” (Exp7, PhTh, 23)*
Relational restructuring	• Early adopters played a pivotal role in motivating peers through a cascading implementation process • The intervention was framed as a meaningful enhancement rather than an additional burden • New forms of interdisciplinary collaboration emerged within and between organizations	*“I only need one person working on it initially, but they can influence at least 10 out of 20 colleagues” (Exp1, PhTh, 98)*
Normative restructuring	• Rehabilitation clinics began seeing themselves as active actors in preventive care • The digital approach required a redefinition of therapeutic roles, particularly regarding boundaries between coaching and psychotherapy • Pilot initiatives fostered innovation and supported collective decision-making around digital integration	*“What I consider essential is that we really redefine our role: What is our role within this digital phase? We are not, and we cannot provide outpatient psychotherapy” (Exp7, PhTh, 13)*
Sustainment (normalization)	• Initial concerns about time intensity and workflow disruption were resolved as processes became more defined • Staff across all levels expressed a clear desire for permanent implementation and broader scaling • The combination of high acceptability, operational functionality, and strategic alignment indicates strong potential for sustainability	*“It has settled in well, and if I look at it now, it’s working normally. The processes are clear. We admit patients every 14 days on Thursday” (Exp2, PhTh, 15)*

Overall, 1.104 segments were coded across the nine interviews. Figure 3 shows the number and relational distribution of coded segments specified for each category following the NPT manual and its domains and constructs. The proportion of coded segments within the domains of implementation mechanisms and outcomes indicates that the topics of resources, purposive actions, creating and adapting existing processes, and the practical effects and consequences of implementing the new intervention were significant to the experts.

### Implementation context

The domain ‘Implementation context’ deals with how the implementation of the intervention is influenced by the organizational and structural environment and consists of four further constructs: *Strategic intentions, Adaptive execution, Negotiating capacity, Reframing organizational logistics*. Detailed results, including all constructs, are available in Supplementary Table 1 in Supplementary Material 4.

The implementation of the intervention and its *strategic intentions* unfolded within a dynamic, multi-layered organizational context shaped by the DRVs strategic principles and long-term goals. The guiding maxim *“Prevention before rehabilitation, and rehabilitation before retirement,”* (Exp10, Admin, 19) served as a strategic anchor. While the clinics and the DRV had extensive rehabilitation experience, the preventive nature of RV Fit Mental Health marked a conceptual shift requiring new approaches to planning and delivery. Overall, the clinics were free to design the intervention within the limits of regulatory constraints. The goal was to offer diverse, individually tailored intervention content to meet participant needs, thereby addressing a previously unmet demand. Among the four constructs of the implementation context, strategic intentions proved particularly foundational: the shared strategic rationale provided by the DRV created a common reference point that guided both the clinics and administrative partners throughout the implementation process, even as practical challenges emerged in the other constructs.

Focusing on *adaptive execution*, the integration of the intervention into existing (clinical) routines proved complex. Established rehabilitation processes required selective adjustments, particularly regarding documentation, communication interfaces, and administrative workflows between the clinics and the DRV. As one expert noted:

*There are adjustments that the rehabilitation clinics still need to make in their clinic software so that we receive the datasets correctly. But these are relatively minor things that should work in the near future* (Exp3, Admin, 49)

Regular meetings, inter-organizational feedback loops, and transparent communication helped manage implementation challenges. Drawing on experience from psychosomatic rehabilitation and other preventive approaches, clinics integrated tested, evidence-based components, including therapeutic modules, theoretical input, and mindfulness exercises.

Nevertheless, the implementation process demanded strong *negotiation capacity* from all stakeholders, especially given the tight 14-day inpatient timeframe. Unlike standard psychosomatic rehabilitation, which typically lasts at least five weeks and allows for a gradual start, the new intervention had to begin immediately. Although resources and professional capacities were in place, this time pressure increased the complexity of planning and execution. The preventive approach also differed therapeutically, relying more on behavioral change support and group settings than on intensive individual psychotherapy. Although it was clearly stated that the clinics already have the “*toolbox anyway*” (Exp2, PhTH, 7), it needs adaptation. Additionally, differences in target populations necessitated improved referral processes and clearer selection criteria. Despite these challenges, administrative integration was largely successful, owing to close cooperation among stakeholders.

Over the course of implementation, organizational logistics were reframed in several ways. Initially, the shift to prevention required structural and procedural realignment, and the absence of established routines made early integration difficult. As the intervention matured, processes stabilized, and it became a fixed, easily plannable element. Therapists and connected staff grew more confident in their roles. Importantly, the preventive approach complemented rather than disrupted existing rehabilitation routines, enabling a broader organizational shift towards embedding prevention within standard healthcare practices. As one expert reflected:

*It’s a fixed program that runs for 14 days. Everyone knows about it. All therapies are planned (…) the entire process is set up as a framework* (Exp2, PhTh, 51)

### Implementation mechanisms

The domain ‘Implementation mechanisms’ contains mechanisms, purposes, and actions, including individual and collective investments, to achieve the goal of implementation. The constructs cover the range of mechanisms and processes of implementation, from the start of planning and conducting the intervention through the (daily) processes of performing the intervention and adapting it, to the step of reflecting (backward/forwards) on the new intervention. The domain consists of four constructs: *Coherence building (‘making sense of it’), Cognitive participation (‘working out participation’), Collective Action (‘doing it’), and Reflexive monitoring (‘reflecting on it’)*. Each is again differentiated with further sub-constructs. Detailed results, including all constructs and subconstructs, are available in Supplementary Table 2–5 in Supplementary Material 4.

The construct *Coherence-building* (‘making sense of it’) aims at the individual and collective sense-making of the new intervention and consists of four sub-constructs: *Differentiation, Communal specification, Individual specification*, *and Internalization*.

The intervention was widely understood as a *differentiation* from previous (rehabilitation) practices at both clinical and administrative levels. The emphasis on prevention rather than treatment stood out as a defining feature, with quicker initiation, shorter duration, less intensive therapeutic engagement, and greater flexibility for participants. The intervention was characterized by quicker initiation, shorter duration, and less intensive therapeutic engagement than rehabilitation. It offers greater flexibility, allowing patients more autonomy through optional (or voluntary) components. Administratively, the intervention was perceived as an organic extension of existing prevention efforts rather than an entirely novel process. At the *individual and communal level*, the intervention was seen as a valuable and timely development despite initial challenges around workflow changes and staff acceptance. Over time, strong *internalization* was evident at both clinical and administrative levels. As one expert reflected:

*The prevention mindset has actually deepened. Of course, I already had that in mind, but it became even clearer to me that, with perhaps not such a HUGE effort, I can achieve quite a lot with selected patients before they even need to go into four weeks of inpatient treatment (Exp2, PhTh, 41)*

Among the four sub-constructs, internalization proved particularly significant—the growing personal and organizational commitment to preventive care was central to sustaining the implementation process. Clinicians expressed a deeper appreciation for preventive approaches and valued the opportunity to achieve significant impact with relatively modest effort. A strong sense of purpose emerged, enabling creative, collaborative work with participants. Co-developing the intervention and seeing patient improvements fostered a strong emotional investment. Administrators also recognized the intervention’s societal relevance and its role in expanding services.

The construct *Cognitive participation* (‘working out participation’) aims at the engagement and collaboration within and between the organizations to conduct and sustain the intervention. It consists of four sub-constructs: *Initiation, Enrolment, Legitimation, and Activation*.

Cognitive participation was driven primarily by the active initiation and involvement of key individuals in leadership positions or with specialized knowledge. These individuals advocated for the new approach, developed and communicated implementation strategies, and fostered the necessary competencies within their organizations. Project managers played a particularly important bridging role between organizations. *Enrolment* within the intervention involved training, continuous reflection, and clarification of roles and responsibilities. While adapting to new ways of working proved challenging for some, many appreciated the opportunity to actively shape an innovative intervention. The long-term benefits of prevention were increasingly *legitimized* and recognized as justifying the implementation effort, and despite the substantial workload, a shared belief in the project’s value *activated* and fostered high levels of commitment. As one expert noted:

*That is why we have certainly put much energy into it, so to speak, so yes. That is why we also see ourselves at the forefront of these things* (Exp2, PhTH, 33)

Among the four sub-constructs, *initiation* proved most prominent—without the sustained engagement of key individuals, the inter-organizational collaboration that characterized the implementation would not have been achievable. Individuals, often holding leadership positions or possessing specialized knowledge, played a central role in initiating and promoting the intervention. They advocated for the new approach, developed and communicated implementation strategies, motivated team members, and fostered the necessary competencies within their organizations. Equally important was building collaborative networks among stakeholders. Effective communication and coordination meetings helped align goals, address challenges, and foster a shared work culture.

The construct *Collective Action* (‘doing it’) refers to joint work to enact the intervention and integrate it into existing structures and processes, and consists of four sub-constructs: Interactional workability, Relational integration, Skill-set workability, and Contextual integration.

Both effective collaboration and practical challenges shaped collective action. A key barrier to *interactional workability* was the inconsistent approach to follow-up care during the digital phase, including the absence of clear organizational tools within the app and difficulties with temporal coordination, as many participants contacted therapists outside regular working hours. At the same time, a structured yet flexible therapeutic framework allowed professionals to individualize care, and feedback mechanisms contributed to trust-building communication. *Relational integration* was well established through clear roles, trust in each other’s expertise, and a shared understanding of the program’s objectives. *Skill-set workability* relied on adapting existing competencies rather than acquiring fundamentally new ones, though therapists supporting the digital phase faced a new dynamic. While close contact in rehabilitation enabled in-depth therapeutic work, the new formats are more focused on shorter, advisory interventions:

*So my role during rehabilitation differs in that I had five sessions and also daily follow-ups […]. This means that the contact was already quite close, and I got to know the individuals and their life situations very well […]. Whereas here, it is more in the direction of psychological counseling or a check-in […].* (Exp6, PhTh, 15)

Regarding *contextual integration*, rehabilitation clinics proved well-suited to host prevention efforts given their existing infrastructure, though challenges arose in aligning the intervention with the diverse needs of the target group. Among the four sub-constructs, *interactional workability* proved most prominent. As one expert put it: *“We have a set therapy program that can be adapted individually” (Exp 7, PhTh, 17).* This balance of structure and flexibility supports workability by providing a clear framework. The inconsistent approach to follow-up care, with varying strategies for appointment scheduling and the absence of clear organizational tools within the app, disrupted seamless communication. This lack of standardization sometimes led to a loss of participant contact. Temporal coordination also emerged as a concern, as many participants are employed and tend to contact therapists outside regular working hours. This strained therapists balancing responsiveness and personal boundaries: *“I’m getting another message now, and I actually have to respond, because I want to maintain that connection” (Exp 8, PhTh, 15*). Nevertheless, where a structured yet flexible therapeutic framework was in place, professionals were able to individualize care. Administratively, processes increasingly functioned smoothly, suggesting growing organizational alignment.

The construct *Reflexive monitoring* (‘reflecting on it’) aims at the individual and collective appraisal of the intervention and its use. It consists of four sub-constructs: *Systematization, Communal appraisal, Individual appraisal, and Reconfiguration*.

The *systemization* and reflection were supported by structured stakeholder communication, which enabled valuable insights into roles, responsibilities, and experiences across technical, legal, administrative, and clinical dimensions. Alongside stakeholder dialogue, scientific monitoring was vital for reflective practice and for uncovering latent issues not immediately visible to practitioners. Structured feedback loops enabled the assessment of the intervention’s impact and the timely, informed adjustment of its implementation. The initial *communal appraisal* was marked by concern over disrupted routines and an increased workload, particularly in the early phases of integration. However, positive participant feedback helped shift these perceptions, and the intervention was increasingly regarded as a meaningful addition to psychosomatic care. In *individual appraisal*, the hybrid format was seen as particularly effective. As one expert noted:

*I see it as a combination of inpatient and digital treatment—fully inpatient is a cost issue, just digital is too anonymous. With this, you can combine these two elements in an ideal way* (Exp2, PhTh, 45)

The *reconfiguration* of the intervention was continuously performed throughout the project in response to feedback, particularly regarding the 14-day schedule and digital aftercare phase:

*For example, some participants have different needs regarding the frequency, intensity, or even the break times. (…) This is something we notice, and it does vary individually among participants (Exp7, PhTh,19)*

Among the four sub-constructs, *Reconfiguration* proved to be the most prominent. Early adjustments optimized the 14-day inpatient phase, focusing on therapy schedules, intensity, and breaks. These individualized considerations prompted a more flexible program design, enabling the intervention to be tailored to a broader range of participant needs. Adaptations also improved scheduling in the digital aftercare phase. Organizational adjustments were necessary as referral volumes increased, requiring revisions to documentation, workflows, and outreach strategies.

### Implementation outcomes

The domain of ‘Implementation Outcomes’ refers to the concrete results and observable effects of introducing and embedding the intervention. It covers how effectively the intervention is integrated into practice and how well it is accepted, adopted, and sustained by individuals and organizations. These outcomes reflect the cumulative impact of the other domains, including the extent to which the intervention aligns with existing workflows, how seamlessly it is incorporated into institutional routines, and whether it achieves the intended benefits for patients and organizations. Ultimately, implementation outcomes provide a lens for assessing the intervention’s success, including efficacy, long-term feasibility, organizational fit, and potential for broader application within the healthcare system. The domain consists of four constructs: *Intervention performance, Relational restructuring, Normative restructuring, and Sustainment (normalization)*. Detailed results, including all constructs, are available in Supplementary Table 6 in Supplementary Material 4.

*Intervention Performance* was central, with the structured 14-day schedule adding clarity, predictability, and improved planning to the clinical routine. The standardized framework enabled streamlined processes, reduced organizational uncertainty, and addressed initial challenges and barriers, with adaptive improvements already made. Technical enhancements and staff training supported the implementation, while positive participant feedback confirmed strong acceptance and sustainment of the intervention:

*Yes, this measure is well to very well received. And that we also, yes, see it as a success for ourselves. Even if it doesn’t apply to 100 percent, but for the majority of participants. So that I would say, ‘Yes, this is a successful measure,’ and I would really say that what should still be kept in mind is the compatibility of the offer or the implementation of the measure with, let’s say, the very different needs of the participants* (Exp7, PhTh, 23)

*Relational Restructuring* required redistributing tasks and responsibilities within existing systems. Strategic communication prevented staff from viewing the intervention as a burden. Instead, it was framed as a meaningful enhancement of existing work and a new innovative approach. The project’s flexible, hybrid design, aligned with care practices and evidence, allowed staff to contribute to and experience the co-creation of the intervention. Crucially, early adopters played a pivotal role in motivating peers, enabling a cascading implementation process:

*This curiosity and gradual involvement help in embedding the innovation within the clinic with minimal effort, provided there is a key responsible person. (Exp1, PhTh, 98)*

Although the project introduces new structures, it aligns with existing tasks rather than completely altering them:

*The challenge is to see how it fits within existing structures. […] It has not fundamentally changed anything; neither has it been a burden nor made things significantly easier. (Exp11, Admin, 47)*

*Normative Restructuring* emphasized the cultural and conceptual shifts in practice. The project introduced digital tools and new working modes, challenging therapeutic routines. Pilot initiatives incubated innovation by exploring brief psychological counselling instead of intensive therapeutic interventions. Most importantly, these changes prompted a redefinition of professional roles, particularly within digital settings, requiring clarity on the boundaries and expectations of care delivery:

*What I consider essential for my colleagues and myself is that we really redefine our role: What is our role within this digital phase? […] That we clearly say: ‘We are not, and we cannot provide outpatient psychotherapy*.' (Exp7, PhTh, 13)

Clinics began seeing themselves as key actors in preventive care, beyond rehabilitation. Enhanced communication structures supported collective decision-making and long-term planning for digital integration, fostering shared responsibility and trust across professional domains.

Finally, *Sustainment* (in the sense of *normalization*) was achieved through gradual integration into clinical and administrative routines. Initial concerns about time intensity and workflow disruption were gradually resolved as the processes became more defined and manageable. Participant admission and seamless daily integration demonstrated normalization. Positive patient feedback and minimal administrative challenges reinforced the perceived success across stakeholder levels, with acceptance exceeding initial expectations. As one expert shared: *“My expectations were actually met and even exceeded. I’m noticing overwhelmingly very positive feedback”* (Exp6, PhTh, 17). Staff across all levels expressed a clear desire for permanent implementation and broader scaling, The combination of acceptability, operational functionality, and strategic alignment indicates strong potential for long-term sustainability, complemented by the understanding that the intervention is still in a phase of active learning and adaptation while important challenges remain.

## Discussion

### Principal results

Using NPT, this study examined the implementation of the new hybrid prevention intervention RV Fit Mental Health. While the implementation was largely successful, particularly under the specific conditions of a model project that afforded greater flexibility than routine care settings. The results showed how the shift from rehabilitation to prevention required adaption at both structural and practical levels and a reorientation and redefinition of mental health care while simultaneously not altering or challenging established structures. Evaluating the implementation, the inter-organizational sense-making, interprofessional collaboration, and iterative adaptation can be identified as key factors. Overall, the intervention led to changes in workflows, professional roles, and organizational identity, while experts showed a strong engagement and appraisal of the intervention contributing to its successful implementation. The intervention addresses several outcomes and integrates inpatient, outpatient, digital and behavioral components which is also reflected within the expert’s perspective. The analysis following the NPT showed the complex implementation recognizing the multitude of re-organizing and creating (established) processes, the variety of adaptation of actions and reactions, and the complex interplay between common daily practices, organizational constraints and external factors.

The implementation of the hybrid intervention RV Fit Mental Health supports the growing relevance of preventive mental health interventions by bridging gaps in early-stage psychosocial care particularly given the challenges and gaps in psychosocial care [57–59]. Especially the contrast to rehabilitation, the prevention intervention opens up new possibilities of mental health care. The results underline how hybrid formats can bridge outpatient and inpatient care while promoting flexibility, autonomy and continuity. As a complex intervention, the intervention integrates inpatient, outpatient, digital, and behavioral elements, targeting multiple outcomes (such as psychosocial well-being, stress symptoms, Health literacy, etc.) [60]. Given the complexity and sensitivity of mental health prevention, our findings underline the necessity for flexible, context-aware implementation evaluation [61, 62]. This is particularly important for the transferability of the results as it has been demonstrated that the success of interventions is dependent on the interaction with the specific context of implementation [63]. These insights may also inform the integration of similar hybrid models across other health domains, particularly those requiring cross-sectoral collaboration. The character of the prevention intervention makes this intervention transferable and comparable to other fields, especially where a strong connection between inpatient and outpatient care, personalization, flexibility and multi-level engagement are necessary [12, 64, 65]. The hybrid approach in this study reflects the growing evidence base for blended care models in mental health. Compared to purely in-person approaches, hybrid interventions offer greater flexibility and continuity of care beyond the clinical setting, while fully digital approaches risk losing the relational benefits of face-to-face contact [66–68]. The hybrid format addresses both limitations by anchoring the digital phase in a therapeutic relationship established during the inpatient stay—a mechanism that emerged as central to acceptance and sustained engagement in our expert interviews. However, as highlighted by Wentzel et al. [68], successful blended care requires careful integration of digital and face-to-face components, clear professional role definitions, and ongoing engagement monitoring, which emerged as key implementation challenges in our study.

The study highlighted how the intervention led to changes in interprofessional collaboration, therapeutic routines and the emergence of new professional identities (e.g. therapists as digital coaches) and thus reflects on broader implications of prevention care and the incorporation of prevention in care routines [69–71]. The implementation process catalyzed a shift in professional roles and individual and organizational norms, illustrating the growing need for digital competencies and flexible care models within clinical and administrative teams in conducting hybrid interventions. Similar changes and challenges in the nature of prevention care delivery by health care professionals are already shown by other studies for mental health and across other settings [21, 72–78]. This changing nature of preventive care delivery is of great importance for further research [3, 5, 79–81]. Despite this reorientation and redefinition within preventive approaches, the results indicate that hybrid interventions can be meaningfully embedded in care routines without replacing or substantially challenging core rehabilitation structures. The insights presented in this study may inform implementation strategies in other fields of mental health care by using hybrid approaches or other fields of prevention, particularly where digital elements must be incorporated. While the present study focused on the expert perspective, the patient perspective on the implementation of RV Fit Mental Health has been examined in two parallel studies within the same project. The pilot study found overall high acceptance of the intervention among participants, while identifying key challenges including the transition between the inpatient and digital phases, the heterogeneity of participants’ diagnostic profiles and symptom severity, and varying levels of eHealth literacy influencing app engagement [82]. These findings are further contextualized by another analysis within the project, in which we identified four distinct psychosocial subgroups among intervention participants with significantly different eHealth literacy profiles, highlighting that digital engagement cannot be assumed to be uniform across the target population [83]. Participants with less favorable psychosocial resources showed lower eHealth literacy, particularly in autonomous app use—a finding with direct implications for the implementation of hybrid interventions. Together, these complementary perspectives underscore that the successful normalization of the intervention from an organizational standpoint, as demonstrated in the present study, must be accompanied by tailored support strategies that account for the diverse needs and digital competencies of participants.

The results following the framework of NPT offered an in-depth-analysis of the complex interplay between the implementation context and the implementation mechanisms shaping the implementation outcomes [35, 36, 39]. NPT offered a systematic lens on the mechanisms within a specific context and its practices to understand how the enacting of the implementation leads to different outcomes and varying degrees of sustainment and workability [36, 39]. The success of the implementation was shaped by its responsiveness to organizational and contextual conditions [63, 84]. The interplay between system constraints and practical workarounds in the clinics and between the other organizations should be highlighted as a dynamic process rather than a static obstacle. The implementation required substantial adaptive work to align with existing structures while establishing new routines in preventive care. The project used its interprofessional and inter-organizational collaboration, which contributed to the normalization of the new preventive practices within traditionally rehabilitation-focused institutions and its localized implementation outcomes. One of the key mechanisms was successful transfer and translation including stakeholder involvement enabling flexibility in planning and iterative adaptation based on feedback and progress. This adaptability is also based on the status of an innovative model project which proved essential benefits in navigating institutional constraints, bridge the inter-organizational gaps and allowed the stakeholders to creatively reframe and align new processes with existing workflows and structures, reflecting the broader utility of adaptive implementation frameworks. The intervention demanded a framework that accommodates non-linearity, context interactions, and layered intervention components [60]. The NPT framework facilitated a systematic understanding of implementation processes, particularly in relation to the incorporation and workarounds in the normalization of the intervention. It helped to analyze key processes, challenges, and adjustments from the perspective of experts involved in planning and implementation, making it especially valuable for qualitative research as it captures how individual and collective experiences shape the adoption of new practices. However, the application of the NPT coding manual also presented challenges, particularly in distinguishing between constructs in ambiguous data segments, as noted in the Limitations section.

The results demonstrated that efficiency and sustainable change are central concerns in hybrid digital interventions and that hybrid intervention require careful integration into care routines without overburdening resources and health care professionals [85, 86]. The complexity of hybrid intervention requires evaluation approaches that capture dynamic interaction with context, multiple target groups and the varying outcomes of the intervention [87–89]. Assessing prevention intervention itself is challenging [90]. The qualitative results presented here underscore the need for process-aligned, user-centered implementation to reduce resource strains [27, 91–93]. Additionally, the insights from this qualitative study presented in this study provide crucial context for interpreting forthcoming effectiveness data. A mixed-methods approach is essential to capture both the qualitative perspectives of stakeholders and measurable dimensions and outcomes of complex health intervention. The triangulation of the research strands is essential in evaluating complex intervention and qualitative insights need to be triangulated with quantitative data on outcomes, adherence, motivation and system impact [94]. The data presented here will be complemented by firstly, quantitative data on efficiency, behavioral changes, adherence and eHealth engagement and secondly, with qualitative data of the perspectives of participants and further analysis of experts (longitudinal approach & focus groups). So, overall, the approach reflects challenges for evaluating the complex hybrid intervention aligning implementation processes, outcomes, and contextual evaluation within a holistic, large scale mixed-methods approach [95, 96].

### Limitations of the study

This study shows several limitations. Overall, in this study, we focused on specific areas of implementation. As we were interested in the inter- and inner-organizational processes, we limited the study to experts who were part of the development and implementation of the intervention and left the participants perspective aside. The results offer valuable insights into early-stage implementation. Further research is needed to validate and extend the results of this study, complementing the experts views particularly through the user perspective and quantitative outcome data. Furthermore, it is essential to assess the long-term sustainability and scalability of the intervention.

Additionally, we would like to highlight the potential of complementary frameworks covering the whole process of developing and implementing interventions. While the NPT captured key organizational and interactional dynamics, complementary frameworks like the CFIR or COM-B, may help address other aspects like user-level implementation barriers or individual behavioral changes [18, 97, 98]. From a theoretical standpoint, as our study is limited to a specific domain of implementation, a more comprehensive and holistic approach also incorporating user perspectives or the steps of the development of the intervention might have provided additional insights. However, future research could benefit from a multi-theoretical approach to also address behavior-specific mechanisms in greater depth. In our case, the results presented here are embedded within a larger scaled multicentric study and will be contrasted by several other sub studies. Within the convergent Mixed-Methods-Design, the qualitative results of the implementation presented here, will be contrasted and complemented by quantitative and qualitative data [96, 99]. The larger scale triangulation of the different data sets of qualitative and quantitative data will be performed in the future – as we also did it for the piloting stage of the intervention [24].

In the context of this study, the use of the NPT for data analysis presents several limitations that should be considered when interpreting the findings. These limitations are primarily related to the fixed coding manual based on the framework developed by May et al. [39]. The manuals’ structure ensured a systematic approach to coding and made the theory more accessible to researchers, especially those less familiar with NPT. By offering concrete definitions and examples, the manual provided a solid foundation that facilitated the analysis of complex qualitative data. However, while the manual’s structure was generally clear, certain aspects of the coding process were difficult to interpret, particularly when the application of specific codes required a deeper understanding of the nuanced theoretical distinctions. This complexity sometimes led to ambiguities in applying the framework, and the application of the codes required significant effort and careful consideration of the data’s nuances.

The reliability and inter-rater consistency of the coding process were overall strengths. The manual’s clear definitions and structured approach ensured that the codes were applied in a consistent manner across different researchers when overcoming initial challenges, which is essential for maintaining the validity and reliability of the analysis. However, despite these strengths, some variation in interpretation still occurred, particularly when applying the codes to more ambiguous or complex segments within the data. Although NPT is an established framework, there were few comparable practical applications in empirical studies available in relation to our research interest – especially for hybrid interventions -, which could certainly have further supported the analysis [33, 34]. In summary, while the coding manual provided a structured and theoretically grounded approach to data analysis, its limitations - particularly in terms of flexibility, depth, and usability - should be acknowledged. Despite these challenges, the manual’s clarity, adaptability, comprehensiveness, and contribution to reliability were significant strengths that supported a rigorous analysis process. Future research could benefit from a more dynamic coding process that allows for greater adaptability to context-specific challenges and the inclusion of additional training materials to support researchers in using the manual effectively.

## Conclusion

This study examined the implementation of the new hybrid intervention RV Fit Mental Health using NPT. It provided an in-depth analysis demonstrating how shifting from an orientation on rehabilitation to prevention required substantial adaptation across clinical/therapeutic, administrative and organizational domains. Overall, the implementation was successful, and the intervention can be described as normalized – while simultaneously being in the state of iterative adaptation. The study revealed key drivers and facilitating factors for successful implementation by stakeholder collaboration and engagement, flexible integration strategies, and context-sensitive evaluation approaches. The intervention RV Fit Mental Health triggered changes and redefinitions of institutional routines, professional roles, and care approaches within its hybrid intervention approach. Thus, this study highlights the value and the benefits and challenges of hybrid interventions. The results emphasize the need for flexible and adapting implementation approaches and underline the relevance of aligning the steps of the development and implementation of new intervention with the realities of delivery contexts. Overall, this study offers transferable guidance for future hybrid (preventive) interventions and contributes to advancing theory-informed implementation research in complex care settings. While the intervention showed promising indicators of sustainability (including growing organizational integration, positive participant feedback, and staff commitment) long-term viability will need to be confirmed in the ongoing evaluation phase of the PE^3^PP project.

## Electronic supplementary material

Below is the link to the electronic supplementary material.

Supplementary Material 1

Supplementary Material 2

Supplementary Material 3

Supplementary Material 4

## Data Availability

The datasets used during the current study are not publicly available due to the privacy regulations of the project but are available from the corresponding author upon reasonable request.

## Abbreviations

mHealth apps: Mobile Health applications
PE^3^ PP: German Acronym for the Project for the development, pilotation and evaluation of a psychosocial prevention intervention
NPT: Normalization Process Theory
DRV: German Acronym for German Pensions Insurance Central Germany
AOK PLUS: German Acronym for Health Insurance Company ‘Allgemeine Ortskrankenkasse’
TUM: Technical University of Munich
COREQ: COnsolidated criteria for REporting Qualitative research
