# Supplementary material for: Using normalization process theory to evaluate the implementation of a hybrid psychosocial prevention intervention in mental health care – a qualitative interview study
Source: BMC Health Serv Res. 2026 May 13;26:700. doi: 10.1186/s12913-026-14631-x (PMC13173747; doi:10.1186/s12913-026-14631-x)
Supplement: Supplementary file 2 — Supplementary Material 2 [file 12913_2026_14631_MOESM2_ESM.docx]

**Additional File 2: COREQ Checklist** (following Tong et al. (2007) [1])

| **Domain 1: Research team and reflexivity** | |
| --- | --- |
| Personal Characteristics | |
| 1. Interviewer/facilitator | Detailed in methods section "Data collection":  Interviewer: Jan Gehrmann (JG), Johannes Stephan (JS) |
| 1. Credentials | JG: M.A.  JS: M.Sc.PH, M.Sc.  Jana Dehner (JD): B.Sc.  Ananda Stullich (AS): M.A.  Matthias Richter (MR): Prof. Dr. rer. soc. |
| 1. Occupation | JG, JS, AS: research associates  JD: Student assistant  MR: Professor (Chair of Social Determinants of Health at TUM), project coordinator |
| 1. Gender | JG, JS, MR: Male  JD, AS: Female |
| 1. Experience and training | Interviewers had significant experience in qualitative research and were led by an experienced researcher (MR). Additionally, they attended external workshops and informal training.  Experience:  JG: Significant experience in qualitative research and interviews, realized qualitative interviews  JS: significant experience in qualitative research and interviews, realized qualitative interviews  JD: experience in qualitative research and interviews  AS: significant experience in qualitative research and interviews  MR: conceptualized and realized several qualitative interview studies previously |
| Relationship with participants | |
| 1. Relationship established | The authors who collected the data or analysed them (JG, JS, JD, AS) had no prior existing relationship with the interviewees. |
| 1. Participant knowledge of the interviewer | Interviewees were informed about the project, interviewer’s educational background and occupational status in advance. Participants had the chance to request further information regarding the provided information. |
| 1. Interviewer Characteristics | The interviewers have a research interest in health services research and (medical) sociology. |
| **Domain 2: Study design** | |
| Theoretical Framework | |
| 1. Methodological orientation and theory | Reported in the methods section "Data analysis". |
| Participant selection | |
| 1. Sampling | Reported in the methods section. |
| 1. Method of approach | Reported in the methods section. |
| 1. Sample size | Reported in the methods section. |
| 1. Non-participation | Reported in the methods section. |
| Setting | |
| 1. Setting of data collection | Reported in the methods section "Data collection":  Interviews: professionals’ workplaces or at home.  Additional information: Interviewees attended the interview either in the workplace or at home. |
| 1. Presence of non-participants | No one else was present besides the participants and the interviewer. |
| 1. Description of Sample | Reported in the methods section. |
| Data Collection | |
| 1. Interview Guideline | The interview guides are described in the methods section "Data collection" and can be found in the Additional Files (see Additional File 3). |
| 1. Repeat interviews | No repeat interview was necessary. |
| 1. Audio/Visual recording | Reported in the methods section "Data collection". |
| 1. Field notes | No field notes were written. |
| 1. Duration | Reported in the methods section. |
| 1. Data saturation | Reported in the methods section. |
| 1. Transcripts returned | Transcripts were not returned to participants. |
| **Domain 3: Analysis and findings** | |
| Data analysis | |
| 1. Number of data coders | Reported in the methods section "Data analysis":  Indexing all interviews and analysing: JG, JS, JD  Summarizing and charting of the indexed data: JG, JS, JD, AS |
| 1. Description of the coding tree | Reported in the methods section "Data analysis". |
| 1. Derivation of themes | Reported in the methods section "Data analysis". |
| 1. Software | Reported in the methods section "Data analysis". |
| 1. Participant checking | Not reported. |
| Reporting | |
| 1. Quotations presented | Different participants' quotes are presented in the results to illustrate the findings with corresponding identifying indication. |
| 1. Data and findings consistent | Yes. |
| 1. Clarity of major themes | Major themes are presented in the results. |
| 1. Clarity of minor themes | As far as the word count permits, we discuss minor themes, too. Additionally, a minor proportion of data fell outside our coding framework. These data either (1) were not related to the scope of the Normalization Process Theory, or (2) focused on the evaluation and optimization of the intervention rather than the nexus of implementation. |
