# Supplementary material for: Using normalization process theory to evaluate the implementation of a hybrid psychosocial prevention intervention in mental health care – a qualitative interview study
Source: BMC Health Serv Res. 2026 May 13;26:700. doi: 10.1186/s12913-026-14631-x (PMC13173747; doi:10.1186/s12913-026-14631-x)
Supplement: Supplementary file 3 — Supplementary Material 3 [file 12913_2026_14631_MOESM3_ESM.docx]

**Additional File 3: Interview guide**

| **Topics**  *NPT Domain or Construct* | **Guiding questions** |
| --- | --- |
| **Context and introduction of the intervention**  *NPT Domain Implementation Context &*  *NPT Construct Coherence (Making sense of it) in the Domain Implementation Mechanisms* | What are your experiences with the newly developed prevention service RV Fit Mental Health?  What are the challenges?  What are the opportunities?  How do your activities as part of the new prevention service differ from your previous activities in the area of rehabilitation?  What were your expectations for the new prevention service? Were your expectations met? If not, why? |
| **Conducting and implementing the intervention #1**  *NPT: Construct Cognitive participation (Working out participation) in the Domain Implementation Mechanisms* | If you look at the entire implementation of the prevention service from the start of the first courses to the present day, how would you rate it overall?  How has the prevention service been received by the participants?  What challenges have you encountered?  What special features are apparent with regard to app use and the digital training phase? (only if therapist and physicians' group)  What are your experiences with the newly developed prevention service RV Fit Mental Health?  What are the challenges?  What are the opportunities?  How do your activities as part of the new prevention service differ from your previous activities in the area of rehabilitation? |
| **Conducting and implementing the intervention #2**  *NPT Construct Collective Action (‘Doing it’) in the Domain Implementation Mechanisms* | How is the measure implemented in their day-to-day work? What special features have emerged or become apparent?  How do you see your role in the continuous implementation of the prevention service (also in contrast to the other departments in the organization)? What demands are placed on you? What demands do you place on the other project stakeholders (DRV, AOK, clinics, possibly scientific support)?  How has your personal understanding of your role changed with regard to your work in the prevention service? (only if therapist and physicians' group)  How is the relationship with the patients? How do you experience the support of the participants during the digital training phase? (only if therapist and physicians' group)  How is the measure accepted and evaluated by your team/organization?  What impact has the new measure had on collaboration in your team/organization? |
| **Reflecting and Outcomes**  *NPT Construct Reflexive Monitoring (‘Reflecting on it’) in the Domain Implementation Mechanisms &*  *NPT Domain Implementation Outcomes* | Overall, when you think about practical implementation, what potential and opportunities lie in the new prevention service?  Where do you still see room for improvement or optimization?  Overall, what were or are the key levers for the successful implementation of the prevention service?  What would you wish for, and what should be the result of the project?  What is needed to make the measure permanent?  Outlook: Apart from the new measure, do you see further advantages in developing a completely new and digital measure? |
| **Closing and Conclusion** | Is there anything else you want to tell us that we haven't talked about yet?  How are you looking forward to the next few months of the project? |
