# Supplementary material for: Using normalization process theory to evaluate the implementation of a hybrid psychosocial prevention intervention in mental health care – a qualitative interview study
Source: BMC Health Serv Res. 2026 May 13;26:700. doi: 10.1186/s12913-026-14631-x (PMC13173747; doi:10.1186/s12913-026-14631-x)
Supplement: Supplementary file 4 — Supplementary Material 4 [file 12913_2026_14631_MOESM4_ESM.docx]

**Additional file 4 - Detailed Results Tables by NPT Domains and Constructs**

*Supplementary Table 1: Results for the Domain* ***Implementation Context***

| **NPT construct** | **Description and main results (including quotes)** |
| --- | --- |
| Strategic intentions | The organizations have a strategic orientation towards rehabilitation so the organizations did not fully meet the needs of the new preventive intervention. *"Well, the topic is new, isn't it? This model project on mental health prevention is new. But ultimately, prevention is not new. It has already been part of our daily work" (Exp11, Admin, 47).*Coordination and adjustments required a shift in thinking from rehabilitation to prevention in order to *"integrate it alongside regular rehabilitation because the process is […] different" (Exp2, PhTh, 16-17).* The new intervention is based on the established Classification of Therapeutic Services (CTS) but the clinics were free to choose so there was potential for adjustments: *"One lever […] is that we design the offer on a content level to meet the goals and content of the participants, that we provide as diverse an offer as possible, also with individual possibilities for customization" (Exp7, PhTH, 33-33).* Overall, the intervention *"fills a large gap in the available offerings" (Exp3, Admin, 445).* |
| Adaptive execution | The integration of the intervention posed a challenge, as the existing rehabilitation processes could not be directly adopted, and specific adjustments were necessary, particularly regarding timelines and administrative requirements (interface between DRV and clinics, decision-making at the DRV, documentation, and billing by clinics for the DRV): *"We are still somewhat in development with regard to documentation. There are adjustments that the rehabilitation clinics still need to make in their clinic software and such so that we receive the datasets correctly. But these are relatively minor things that should work in the near future" (Exp3, Admin, 48-49)* This required specific adjustments and flexibility to integrate the new practices into daily workflows. *"We quickly realized that this is not running smoothly, but over time, as we have now noticed, increasingly small problem areas have popped up" (Exp2, Admin, 3).* At the same time, the implementation drew on existing processes of established rehabilitation and other new already implemented preventive measures of the DRV or other model projects. Regarding the intervention contents, no disruption was experienced: *"The elements, introduction to mindfulness, mindfulness exercises, etc., are all relatively identical to what is in rehabilitation" (Exp1, PhTh, 24).* Overall, the intervention could be carried out as planned and *"integrates quite normally" (Exp1, PhTh, 50).* The project stakeholders were able to integrate a successful form of communication to address these challenges and increase the efficiency of decision-making processes. Regular feedback, meetings, and coordination both within the organizations and inter-organizationally made it possible to identify and solve problems early on. |
| Negotiating capacity | The implementation posed overall challenges but as one interviewee explained, *“we have the toolbox anyway, right? So, we have all the colleagues on board, all the possibilities” (Exp2, PhTH, 7*). Despite having the necessary resources and support, it remained challenging to implement the program within the brief 14-day timeframe. Unlike the usual rehabilitation process, where a few days are allocated for the initial setup, the intervention workflow had to be activated immediately. Another distinction between prevention and the more intensive psychotherapeutic work in rehabilitation was the type of therapy offered. As one respondent noted, *"In rehab, it's the case that despite many therapies being offered in group settings, individual psychotherapy is seen as something very essential, and even though it's not like outpatient psychotherapy, it's still more intensive, with therapy goals and individual topics being worked on" (Exp7, PhTH, 15).* In contrast, the preventive approach did not provide the same depth, making it harder to tailor the interventions to specific needs. Additionally, the target groups are different, requiring more precise patient selection and referral processes. One interviewee pointed out the importance of effective referral criteria, saying, *“Referral criteria need to work, otherwise, it’s really, really difficult” (Exp2, PhTH, 49).* |
| Reframing organizational logistics | Prevention has become a more prominent focus in rehabilitation clinics. As one interviewee noted, *“I think yes, prevention has come to the forefront, because it wasn’t as present in our clinic before... and now prevention seems to have a greater importance in the clinic.” (Exp 2, PhTH, 29). For* the implementation of the new intervention, significant adjustments to organizational logistics were necessary, with processes needing to stabilize and become routine. Initially, the integration of preventive interventions into existing workflows was challenging. *“The processes had to settle and become routinized, so everyone knows what they need to do. Until then, it was a difficult transition period, where this feeling of security... this sense of routine had to be established.” (Exp 8, Admin, 39)* Over time, however, the routine process began to solidify, and a consistent structure emerged, allowing the program to run smoothly. As another interviewee highlighted, *“It’s a fixed program that runs for 14 days. Everyone knows about it. All therapies are planned, and the patient arrives (...) the entire process is set up as a framework, with only minor changes depending on the patient’s symptoms or other factors.” (Exp 2, PhTh, 51)* In terms of content, the new preventive intervention addresses a gap in the rehabilitation offerings, catering to a specific need that had not been met previously. As one respondent pointed out, *“It really fills a gap in normal prevention, which we didn’t have before.” (Exp 11, Admin, 9).* Overall, the integration of the intervention did not fundamentally alter the routine in rehabilitation work itself. One interviewee explained, *“So, when I work on rehabilitation, it’s simply something I keep in mind, and... so in everyday work, nothing has really changed in that sense.” (Exp 11, Admin, 33).* |

*Supplementary Table 2: Results for the Construct Coherence Building in the Domain* ***Implementation Mechanisms***

| **NPT sub-construct** | **Description and main results (including quotes)** |
| --- | --- |
| Differentiation | The intervention is perceived as distinctly different from previous practices as the most significant difference is the overall focus on prevention. The intervention begins more quickly and lasts for a shorter period than traditional rehabilitation, which requires a more structured program. One clinic employee remarked, *"A big difference from rehabilitation is that we don’t go as deep, but it can be easily integrated into the clinic" (Exp1, PhTh, 26).* Furthermore, the intervention involves less intensive psychotherapy and places a stronger emphasis on prevention and counseling which is also reflected in the training phase, which, through its digital format, differs in both intensity and duration from traditional rehabilitation programs. The digital follow-up allows for longer-term engagement and continuous feedback, *"In rehabilitation, we would not see the participants again after five weeks. With the digital phase, we get direct feedback and can see what changes in everyday life" (Exp7, PhTH, 17).* Another key difference lies in the flexibility allowed during the intervention. As one clinician mentioned, *"We give the patients more autonomy and offer optional or voluntary activities, like consultation hours, which some participants use, while others appreciate the free time" (Exp7, PhTH, 17).* On the administrative level, the intervention is seen as new approach involving considerable effort to ensure compatibility with existing processes such as recruitment, documentation, and technical requirements. A representative from the administrative side stated nonetheless, *"A bit more work at the beginning, but overall, it’s not a big effort" (Exp10, Admin, 29).* |
| Communal specification | The integration of prevention into rehabilitation clinics represented a fundamental shift in focus on structural and operational levels also requiring additional effort: *“You just have to keep an eye on the other burdens, right? (...) to be aware of the workload (...). But otherwise, it’s a good thing, and I think the clinic is generally well positioned for it, with the offerings we already have within our standard rehabilitation services.” (Exp 2, PhTH, 7)* The initial integration into existing workflows required careful coordination, and early involvement of all key stakeholders accompanied by initial worries: *“At first, there was maybe some uncertainty about how the workflows could be integrated, and it had to find its place because it was quite ambitious to schedule the program so tightly in terms of time.” (Exp 2, PhTH, 15)* Ensuring acceptance among colleagues was a key step, as the intervention disrupted established processes. One interviewee described this challenge: *“First, acceptance among colleagues has to be created. As I mentioned before, you’re interfering with established routines (...). Now, you have to talk to therapy planning, then to patient management (…), and to the nurses.” (Exp 8, PhTH, 39)*  A major success factor in this transition was a shared understanding between different organizational units, both on the clinical and administrative sides. Continuous evaluation of participant allocation and the ability to adapt to feedback played a crucial role in refining the process. From a therapeutic perspective, working on preventive care was seen as both meaningful and varied. *“I think it’s also a great opportunity for employees to have a varied job. Sometimes they are fully involved in inpatient care, sometimes they focus more on the training phase. (…), it’s a diverse and exciting job where you can really shape things.” (Exp 6, PhTH, 41)* Ensuring that prevention was distinct from traditional rehabilitation work was essential. *“When the group dynamic is right, working with participants is a very rewarding experience... it extends beyond the usual scope of making positive changes. Colleagues who have chosen to work in the prevention project generally say, ‘Yes, this is exactly what I want.’ But there are also those who prefer to work more intensively in psychotherapeutic rehabilitation and choose to stay in that field.” (Exp 7, PhTH, 21)* On the administrative side, the prevention program was well integrated into existing structures and the processes that had been developed in theory proved to be highly functional in practice: *“That’s exactly how we planned it - to minimize disruptions as much as possible.” (Exp 10, Admin, 11)* |
| Individual specification | The implementation was perceived as challenging, yet it is seen as a beneficial development. One expert acknowledges the difficulties but emphasizes the long-term advantages of prevention: *"It is certainly not easy to implement this product, but in the long run, it is scientifically proven that prevention helps keep people healthier for longer, preventing early rehabilitation or even premature retirement. The path taken here is the right one." (Exp 10, Admin, 25).* The overall structure and methodology of the program are regarded as particularly valuable and beneficial to ensure sustained motivation and engagement: The consistent presence of the same therapist over an extended period is viewed as a crucial factor in maintaining participant motivation and ensuring successful engagement. However, some therapists describe their role as being more general rather than deeply therapeutic. Despite this, therapists report the rewarding nature of working with participants who are not as severely burdened as those typically encountered in rehabilitation settings: *"It is incredibly rewarding to see the positive feedback and to work with individuals who are generally less burdened than those encountered in rehabilitation. The nature of the work and the working conditions are both enjoyable." (Exp 6, PhTh, 34)* Nevertheless, there is some uncertainty regarding participant engagement over the full course of the project. One expert expresses concerns about whether participants will adhere to the program in the long term: *"We don’t yet have data on long-term participation. The two-week inpatient phase works as expected, but the real question is: How many continue for the full twelve weeks? And even beyond that, do they truly feel healthier? Measuring the success of prevention from the patient’s perspective remains a challenge." (Exp 1, PhTh, 30)* |
| Internalization | Both clinics and the administrative level demonstrated a strong internalization of the intervention, attributing significant (societal) value. The value placed on prevention and early intervention was emphasized as being a meaningful and relatively low-effort way to achieve significant results in improving patients' health. One participant reflected, *"The prevention mindset has actually deepened. Of course, I already had that in mind, but it became even clearer to me that, with perhaps not such a HUGE effort, I can achieve quite a lot with selected patients before they need to go into four weeks of inpatient treatment" (Exp2, PhTh, 41).* This indicates that the intervention was successfully internalized, enhancing the staff's appreciation of its impact in reducing the burden of severe illness. It was also appreciated to work closely with patients and participate in the development of the intervention. One participant described it as a *"heart project"* that allowed for visible changes and collaboration with the patients: *"It’s a heart project because being able to design something with the participants, to see changes, and to co-create something together, especially in collaboration with everyone, is something really valuable." (Exp7, PhTH, 35).*  At the administrative level, the DRV and AOK also recognized the value of the intervention and its significance for expanding their service offerings. One expert described the intervention’s importance for their own work, stating, *"Of course, it’s additional work to coordinate with colleagues, keep track of everything, participate in meetings, and so on." (Exp5, Admin, 47).* This reflects an acceptance of the increased workload, acknowledging the intervention's importance and value despite the additional effort required. One expert reflected on the initial uncertainty, saying, *"It was a pilot project, and expectations were, at first, just: 'Let’s see what comes out of it, how it’s accepted by the insured.' This was something completely new, something we had never offered before. And for that, I find the response rate quite impressive, that it’s been accepted by the insured" (Exp11, Admin, 17).* |

*Supplementary Table 3: Results for the Construct Cognitive Participation in the Domain* ***Implementation Mechanisms***

| **NPT sub-construct** | **Description and main results (including quotes)** |
| --- | --- |
| Initiation | The implementation of the intervention was crucially driven by key individuals within the involved organizations*: "That is why we have certainly put much energy into it, so to speak, so yes. That is why we also see ourselves at the forefront of these things" (Exp2, PhTH, 33).* Their tasks included promoting the planned preventive service, developing and publicizing the implementation strategies, and motivating and fostering the necessary competencies of the teams involved in implementing. The successful implementation depended on effective collaboration between various stakeholders and project participants (both internally within the DRV MD in joint sessions involving project management, case processing, legal department, etc., and within the project framework between the DRV MD, clinics, health insurance companies, and scientific support). This created a unified understanding and shared work culture. In this context, the project management was particularly a key person who systematically promoted networking and exchange. *"I am, of course, also the link to the specialist department, just as much together with my colleague. And here, it is naturally always important to present clearly what we are accomplishing, where adjustments might be necessary.” (Exp3, Admin, 35).* |
| Enrolment | The process of how experts were involved in the intervention included raising awareness, training, and clarifying expectations and achievable goals. Clear roles and responsibilities were defined to ensure a smooth process. *"The colleagues are also firmly integrated, those who run the program, so yes. We have a physiotherapist who is firmly integrated into this, (…) right? Also, the psychological colleagues are increasingly involved for admissions, discharges. The physicians had to be trained" (Exp2, PhTh, 15).* Since the intervention was new, a necessary culture for implementation was created, and relevant organizational structures were established. *"The concepts have to be drafted, the treatment plans have to be created, and the whole management around it has to function so that I can admit them, care for them, and ensure their continued support and treatment. That all has to be arranged and organized first" (Exp5, Admin, 35).* The acceptance of the intervention varied. While many employees appreciated the opportunity to actively participate in an innovative model project, adapting to new working methods proved challenging. *"This great opportunity to be able to co-create; also to have some room for maneuver in shaping the measure. (…) this opportunity, that is really unique" (Exp7, PhTh, 43).* However, employees recognized the chance to be pioneers in preventive services. *"The feedback I have so far from the colleagues […] they enjoy working in the prevention project. So the colleagues who have chosen to do this usually also say: 'Yes, this is exactly what I want.'" (Exp7, PhTh, 21).* Collegial support and positive feedback contributed to further motivation and a positive perception of the project. |
| Legitimation | The implementation was perceived as challenging, yet it is seen as an essential and beneficial development. One expert acknowledges the difficulties but emphasizes the long-term advantages of prevention, particularly in maintaining health and avoiding early rehabilitation or retirement: *"It is certainly not easy to implement this product, but in the long run, it is scientifically proven that prevention helps keep people healthier for longer, preventing early rehabilitation or even premature retirement. The path taken here is the right one." (Exp 10, Admin, 25)* One expert highlights the role of an app-supported training phase in increasing accessibility and choice for participants: *"This is an important component in the prevention landscape, especially concerning the range of services before rehabilitation begins. We also have an app-supported training phase with therapeutic guidance, which I find particularly beneficial. If this service is established and offered by other pension insurance providers, it will significantly enhance the right of choice for participants. More clinics offering app-supported training means a much broader selection, as location is no longer a limiting factor." (Exp 3, Admin, 15)* The overall structure and methodology of the program are regarded as particularly valuable and beneficial. One expert praised not only the early involvement of participants but also the way facilities have structured the program to ensure sustained motivation and engagement: *"I think the approach we have chosen—informing insured individuals early and involving them in the program—is very good. The content, the way facilities structure and implement the concept, and the continuous support from the same therapist for six months are all very positive aspects. Participants can only benefit from this." (Exp 5, Admin, 33).* |
| Activation | Designed to offer preventive care for employees with psychological diagnoses, reaching the right participants remains a challenge, as different recruitment strategies attract individuals with varying severity levels and stages of illness. One expert highlights the complexity of this process: *"Our expectation was and still is to offer a high-quality service that meets the needs of employees with psychological diagnoses. That’s why we developed this preventive service for this specific target group. A challenge remains that we use different recruitment strategies, reaching participants with varying severity and stages of their condition. This is something we continue to evaluate and discuss." (Exp 3, Admin, 25).* A key aspect of successful activation is structuring the digital training phase in a way that maximizes motivation and ensures effective therapeutic support. While flexibility is needed to accommodate individual needs, fixed appointments provide a necessary framework: *"That’s why clear agreements are essential. They provide security for both me and the participants. I schedule the coaching sessions in advance for weeks 4, 8, and 12 to create a structured process." (Exp 8, PhTh, 29)*  Further improving recruitment pathways is seen as essential for the long-term success of the program. Experts emphasized the value of scientific monitoring and external feedback to refine the approach and identify potential areas for improvement: *"It’s an opportunity to collaborate and develop the program further with participants. Regular feedback from scientific research would be invaluable to identify necessary adjustments or hidden challenges that participants might not explicitly mention." (Exp 7, PhTh, 23)* The activation process has also required significant collaboration among different stakeholders. One expert acknowledges both the constructive cooperation and the high workload: *"Everyone has contributed constructive ideas to ensure proper implementation, particularly regarding access through the pension insurance system. It has been remarkable, but also an enormous effort—one that we cannot sustain at this level with our regular staff." (Exp 11, Admin, 19)* |

*Supplementary Table 4: Results for the Construct Collective Action in the Domain* ***Implementation Mechanisms***

| **NPT sub-construct** | **Description and main results (including quotes)** |
| --- | --- |
| Interactional workability | The intervention integrates into existing workflows and interaction routines to varying degrees although the new processes gradually became functional and routinized. A key barrier is the inconsistent approach to follow-up care among therapists. This lack of standardization led, in some cases, to the participants losing contact with the program during the digital training phase. This illustrates how interactional workability is impaired when there are no clear or coordinated procedures in place. Technical and organizational limitations also negatively affect the quality of interactions as for instance, there is no proper organizational tool in the app. Another issue is temporal coordination. Since many participants are employed, feedback and appointment requests often come in the evenings. As one expert put it: *“I’m getting another message now, and I actually have to respond, because I want to maintain that connection” (Exp 8, PhTh, 15*). At the same time, there are examples of well-functioning interactional structures. One expert emphasized: *“We have a set therapy program that can be adapted individually” (Exp 7, PhTh, 17).* This balance of structure and flexibility supports workability by providing a clear framework while allowing personalization based on participant needs. Another essential element for workability of the intervention is the feedback loop with participants. One expert explained: *“We receive regular feedback from patients, we see where someone is at, and we can intervene effectively” (Exp 7, PhTh, 11)*. Feedback is actively used to improve interaction - indicating a high level of interactional workability. This is also reflected in the reliability of communication, which builds trust: *“That gives me a sense of security and gives the participant a sense of security” (Exp 8, PhTh, 29).* Workability of the intervention is further demonstrated in the ability to respond flexibly to individual needs. Finally, administrative processes appear to be working well. |
| Relational integration | The implementation of new projects and innovative services within existing structures requires the redistribution of workloads and responsibilities. This necessitates a clear communication strategy to ensure that staff perceive these changes as manageable rather than merely additional work: *"So we had to say a few times at the beginning that we currently have a few fewer rehabilitation patients, so we have already made some adjustments; that it is not just extra work." (Exp1, PhTh, 64).* The integration of new services into the framework of a collaborative project allows for greater flexibility and involves aligning new interventions with existing guidelines and scientific evidence while also incorporating professional experience: *"I believe the advantages are that we are always aligned, for example, with the guidelines of the German Pension Insurance for preventive measures, or with scientific research findings. This gives us the opportunity to co-develop and design services, bringing in our own ideas and practical experience. We can then assess the effectiveness based on direct feedback and make necessary adjustments. That is something we normally do not have to this extent in rehabilitation, and we also have greater flexibility in designing the intervention” (Exp7, PhTh, 37).* |
| Skill-set workability | In implementing the intervention, specific tasks were distributed among various professionals based on their skills and availability. *"That is why everyone has to know: Who fills out what, and where? These things were all uncharted territory for us, but it has leveled out well, and when I look at it now, it is running normally. The processes are also clear" (Exp2, PhTh, 15).* A key component of successful implementation was the dissemination of information among the involved stakeholders. Physicians, therapists, administrative staff, and other professionals had to familiarize themselves with the new procedures and requirements. The individuals involved, whether in approving prevention by the administrative side or in its implementation in the clinics, did not need to learn fundamentally new skills but had to adapt their existing skills to the new processes of the intervention. Challenges arose mainly among the therapists who supported the digital training phase, particularly in adapting the preventive approach, managing their work schedules and scheduling digital coaching sessions. Unlike the work in the rehabilitation area, this contact was not deeply therapeutic as the therapists functioned merely as support and monitoring person meant to (in)directly induce behavioral change. Additionally, a particular dynamic emerged newly concerning proximity and distance in the therapeutic relationship due to the sole digital connection via app and video calls. |
| Contextual integration | A challenge was to align the needs and expectations of the heterogeneous target group with the requirements of the intervention. Prevention and rehabilitation participants encounter each other daily at the clinic, whether in the cafeteria, while smoking, or in other areas. The prevention participants are not perceived as outsiders by the administration and the therapeutic staff but experience equal treatment, which promotes seamless integration: *"But what we definitely have here, since we have the tools here with us, therefore yes, from that point of view, this rehabilitation clinic is ideally suited. In general, I believe rehabilitation clinics are ideally suited for prevention because they have everything on board" (Exp2, PhTh, 43).* The intake of prevention participants by the DRV leads to challenges, as occasionally, people were admitted to the program who were not optimally suited for it. It was recognized in the clinics that prevention participants have different needs than rehabilitation participants, which required a shift in thinking and the integration of the prevention concept within the organizations, a shift that was successfully anticipated. *"I think prevention has moved more into the foreground because in the past, I believe, it was not so present in our clinic. Sure, prevention was known, etc., but I believe that now it is being updated a bit more since we are now actually implementing it ourselves, so that even the colleagues, even those who are not involved in prevention, so to speak, still have it on their radar a bit, and thus prevention might have a greater significance in the clinic than it might have had before" (Exp2, PhDh, 29).* |

*Supplementary Table 5: Results for the Construct Reflexive Monitoring in the Domain* ***Implementation Mechanisms***

| **NPT sub-construct** | **Description and main results (including quotes)** |
| --- | --- |
| Systematization | Access to information about the effects of the intervention and its components was primarily facilitated through close collaboration and communication among the involved parties. The project structure itself enabled regular exchanges, allowing participants to gain insights into each other’s work and processes. One expert stated, *"I was very pleased that I was able to identify suitable experts from the various fields and that they were willing to collaborate (…) I see this as a tremendous benefit" (Exp3, Admin, Pos. 29).* This interdisciplinary cooperation, covering technical, legal, and administrative aspects, played a vital role in the successful development of the project. Another key point that was repeatedly highlighted was ongoing communication with key stakeholders, particularly in addressing problem areas. One interviewee shared their experience of continuous discussions among stakeholders, which helped identify and resolve challenges promptly. They explained, "*We are still in discussions with the DRV (…) regarding problem areas and other matters, so things are going quite well in that regard, I think. And, as I mentioned, this consultation with the medical service is also ongoing" (Exp2, PhTh, 55).* Additionally, the role of scientific monitoring and continuous evaluation was emphasized. Experts expressed that regular external feedback was essential for the project's development. One participant pointed out, *"We are always interested in scientific feedback, which we have already received to some extent. However, having regular feedback from scientific monitoring would be beneficial. (..) Are there any necessary changes? Are there critical points that perhaps the participants have not mentioned directly but have emerged in hindsight?" (Exp7, PhTh, 23).* |
| Communal appraisal | Overall, initial apprehension was particularly driven by the fact that the intervention required integration into pre-existing routines, which caused stress for many staff members. As one expert described, *"at first, it was quite a lot, I mean, there had to be acceptance among the colleagues... as I mentioned before, it involves interfering with established processes, and that stressed many people." (Exp8, PhTh, 39).* This highlights the initial resistance to change that often accompanies the implementation of new interventions in healthcare settings. However, despite this initial hesitation, the positive feedback from patients and the developing and implementing new approaches significantly contributed to the appreciation of the intervention. As one participant noted, *"Those who have chosen to work in the prevention project generally say, 'Yes, this is exactly what I want to do.' But there are also colleagues who say, 'I would rather work more intensively in psychotherapy,' and then they say, 'Thank you for the offer, but I would just prefer to stay in rehabilitation care'" (Exp7, PhTh, 21).*  The intervention was seen as a valuable supplement to psychosomatic care, and there was strong interest and support for the progress of the project and the app being used. One interviewee stated, *"I have the impression that it is very positively rated. There is a great interest in knowing about the project's progress and also in the app being used, about the content being delivered. Yes, there is a great interest and support for the project. That’s the perception here" (Exp3, Admin, 41).* The intervention was also seen as filling an important gap in available services. As one expert pointed out, *"I think our preventative service fills a major gap. There are so many employees with mental health diagnoses, and if we can reach them earlier, we can prevent repeated or prolonged sick leave, or even avoid the need for rehabilitation" (Exp3, Admin, 45).* |
| Individual appraisal | Many participants believed that the intervention could significantly impact the health and work ability of patients, which, in turn, could have both personal and societal benefits. The ability to engage patients early on and provide support through a combination of personal care and digital tools was seen as ideal. One expert explained, *"Well, as I said, I see it as a combination of, let’s say, inpatient and digital treatment, so to speak, because I think that’s the ideal combination. Because you can do just one of them, but it* *has clear disadvantages. Fully inpatient is definitely a cost issue, so yes, I don’t think it’s something that should be offered on a large scale, or even to many patients. Just digital is too anonymous for me sometimes, so yes, where you can’t push things, (…) And with this, you can combine these two elements of therapy in an ideal way.” (Exp2, PhTh, 45).* Moreover, feedback from participants indicated high satisfaction with the intervention, reinforcing its perceived value. One interviewee noted, *"Then with the satisfaction of the participants, I think it is still high. So when the participants have gone through the program, I experience many satisfied people, and many who also describe subjectively that they felt really good" (Exp8, PhTh, 9).* This satisfaction is a critical indicator of the intervention’s success, further supporting its long-term potential. The intervention was also seen as offering significant opportunities for all involved parties. It was viewed as particularly beneficial for the insured individuals, enabling them to address stress resilience, mindfulness, and self-care early on, which could prevent prolonged sickness leave, illnesses, and related impairments. As one expert stated, *"I see the whole project as offering very many opportunities for everyone involved. For the insured person themselves, that they start doing something for themselves early on in terms of stress resilience, mindfulness, and self-care, thereby preventing the onset of prolonged sickness and diseases, and also the associated impairments. This means that the psychological health of the insured person is restored much more quickly and also maintained in the long term" (Exp5, Admin, 75).* |
| Reconfiguration | Continuous adjustments and adaptations were made throughout the project to ensure that the intervention met the needs of both patients and the involved staff. These adjustments primarily focused on optimizing the program structure and the design of the 14-day initial phase, which was regularly discussed and refined. One expert pointed out the need for individual adaptations based on patient preferences: *"For example, some participants have different needs regarding the frequency, intensity, or even the break times. There are participants who say, 'Yes, the therapy program is optimal as it is, I’m taking the maximum from it.' And then there are participants who say, 'Wait, I would prefer a 30-minute or 45-minute break or even an hour’s break between the therapies. I would prefer fewer therapies.' This is something we notice, and it does vary individually among participants" (Exp7, PhTh,19).* Additionally, the process of adapting the aftercare scheduling in the digital phase became more flexible, allowing better accommodation of both the patients’ and healthcare providers’ needs. The integration of new technologies, such as WhatsApp groups, was also introduced to encourage more active communication among participants. One expert noted the shift towards digital support: *"Further, as we already discussed in the focus group interview results, there will be a discussion about whether fine adjustments can still be made, especially concerning the organization and process of our prevention program. I’m particularly thinking about the weekdays: Which weekday should we start or end the inpatient phase?" (Exp3, Admin, 17).* In response to the short times for patient referrals from the DRV, more participants were invited, which required further organizational adjustments including revising processes and documentation to accommodate the increased demand. One expert described: *"Well, it has become clear that the development of processes, including all the documents, forms that had to be revised, flyers, and such, is quite an effort. And we then found a good approach by not starting the different recruitment strategies in parallel, but instead doing it gradually once the development was complete" (Exp3, Admin, 33).* This stepwise approach helped manage the logistical challenges that arose as the project progressed. |

*Supplementary Table 6: Results for the Domain* ***Implementation Outcomes***

| **NPT construct** | **Description and main results (including quotes)** |
| --- | --- |
| Intervention performance | Introducing a schedule for the entire intervention led to a clear structuring of processes. This enabled better planning and predictability in the clinic's daily routine. *"The program is basically set, so yes, it is a fixed program that runs for 14 days. All the colleagues are informed. All therapies are planned, and the patient does not arrive like usual [for rehabilitation], where the therapist first looks to see what therapies, they could join. You plan a bit. A fixed program, so to speak. The entire process is set as a framework, so to speak, and there are only minor changes depending on the patient's symptoms or other things, but otherwise, the program is fixed for everyone involved and therefore, of course, nicely plannable" (Exp2, PhTh, 51).*  Additional resources, such as technical equipment and training, were provided to maximize the efficiency of the intervention. The positive feedback from the participants shows a high level of acceptance for the intervention. *"Yes, this measure is well to very well received. And that we also, yes, see it as a success for ourselves. Even if it doesn't apply to 100 percent, but for the majority of participants. So that I would say, 'Yes, this is a successful measure,' and I would really say that what should still be kept in mind is the compatibility of the offer or the implementation of the measure with, let's say, the very different needs of the participants" (Exp7, PhTh, 23).*  Despite the positive feedback, there were also challenges, particularly concerning coordination and communication between the various stakeholders, which could be managed through regular exchange. Regular meetings to exchange inter-organizationally continue to be conducted. *"So, I would say we work well together here and discuss the problems. We don't always, right away, all have the same opinion. That wouldn't be normal either, yes. But in the end, we can always agree on a good way, I think. I am very satisfied with the project group and the project work" (Exp10, Admin, 35).* |
| Relational restructuring | The establishment of new technical infrastructures, such as digital workspaces, plays a key role in the adaptation of the intervention: *"Employees need to understand that this might be a meaningful addition, making it worth investing an extra hour now to push the project through and establish the necessary technology. In the end, this led to the creation of a computer room with multiple screens instead of just one per patient. The ultimate goal is that every patient receives a tablet upon admission, containing everything they need—meal plans, schedules, yoga exercises, autogenic training, and health lectures. That is my vision of rehabilitation." (Exp1, PhTh, 64).*  The long-term establishment of innovative care models within clinics depends on a cascading implementation process. A key principle is that one committed professional can inspire broader participation, gradually integrating the new approach into everyday clinical practice: *"That is exactly the advantage of such projects. I only need one person working on it initially, but they can influence at least 10 out of 20 colleagues, who might step in occasionally, observe the process, and hear from patients about their experiences. This curiosity and gradual involvement help in embedding the innovation within the clinic with minimal effort, provided there is a key responsible person." (Exp1, PhTh, 98).*  New forms of collaboration also emerge within organizations, enhancing alignment and optimizing processes beyond the project itself. Although the project introduces new structures, it aligns with existing tasks rather than completely altering them. This highlights the integration of innovations into routine operations without significantly increasing workload or complexity: *"Well, the topic is new, right? This model project on mental health prevention is something new. But ultimately, prevention itself is not new—it has always been part of our daily work. The project simply emphasizes mental health specifically. The challenge is to see how it fits within existing structures. [...] It has not fundamentally changed anything; neither has it been a burden nor made things significantly easier." (Exp11, Admin, 47).* |
| Normative restructuring | The introduction of new technologies and working methods plays a central role in the process of normative restructuring. Pilot projects serve as an important driver of innovation to test new ways of working: *"Well, only through pilot projects can innovation be introduced somewhere. Otherwise, the pension insurance operates within its standard business in the areas of rehabilitation, prevention, and pensions. That's why pilot projects are needed to try out new approaches, test new services, and explore new forms of collaboration with other specialist areas." (Exp 3, Admin, 63)*  Another key aspect of normative restructuring is the adaptation of care roles. Rehabilitation clinics are increasingly seen as key players in preventive care: *"because we have the tools here with us, therefore, yes, from that perspective, this rehabilitation clinic is ideally suited.” (Exp 2, PhTh, 43).* At the same time, work dynamics in therapeutic contexts are changing, as reflected in altered workflows. The restructuring becomes particularly evident in the differentiation between traditional therapy approaches and new counseling models. While close contact in rehabilitation enabled in-depth therapeutic work, the new formats are more focused on shorter, advisory interventions: *"So my role during rehabilitation differs in that I had five sessions and also daily follow-ups [...]. This means that the contact was already quite close, and I got to know the individuals and their life situations very well [...]. Whereas here, it is more in the direction of psychological counseling or a check-in [...]." (Exp 6, PhTh, 15).*  This requires a clear redefinition of therapeutic roles to set clear boundaries and expectations for both professionals and patients: *"What I consider essential for my colleagues and myself is that we really redefine our role: What is our role within this digital phase? [...] That we clearly say: 'We are not, and we cannot provide outpatient psychotherapy.'" (Exp 7, PhTh, 13).* Another aspect of normative restructuring concerns interdisciplinary collaboration and decision-making. New models and structures create opportunities for improved care. This contributes to the long-term implementation of digital processes: *"So hopefully, it [the intervention] will function in the long term as a kind of door opener. That’s a good term." (Exp 2, PhTh, 68).* |
| Sustainment (normalization) | The implementation of the intervention in the clinics was largely successful. Despite initial concerns about additional time requirements, these were alleviated as clear processes and workflows were established. One expert reflected, *"But it has settled in well, and if I look at it now, it’s working normally. The processes are clear. We admit patients every 14 days on Thursday. So, it has integrated into the system" (Exp2, PhTh, 15).* This highlights the successful integration of the intervention into the clinic’s regular operations, illustrating the normalization of new practices over time.  Furthermore, positive feedback from patients reinforced the perceived success of the intervention: *"in general, it has proven itself in the sense that we noticed that the patients accept it well. Also, our therapists […] have received initial approval from all sides or have noticed that it simply works well" (Exp2, PhTh, 3).* This acceptance across different stakeholders underlines the intervention’s sustainability. The acceptance of the preventive measure by patients was perceived as high, with positive responses exceeding expectations. One expert shared, *"my expectations regarding prevention and how it works were actually met and even exceeded. I thought that with so few one-on-one sessions in the training phase and so much independence in using an app, it might be less utilized overall or less effective. But I’m noticing overwhelmingly VERY positive feedback, much gratitude for the measure, and yes, my expectations have been exceeded" (Exp6, PhTh, 17).* This highlights the endorsement of the intervention, suggesting that it is not only sustainable but also highly appreciated by participants.  At the administrative level, the implementation was also seen as largely successful, despite initial challenges in participant access and recruitment. One expert explained, *"What’s also pleasing is […] that our (…) department, which has to implement it, reports no issues. I have to put it this way: they have no problems with the organization. We thought, well, 'this is new, and there will be some hiccups here and there, and there will be complaints.' But no, things are running smoothly. So, the theory we developed has turned out to be quite applicable in practice" (Exp10, Admin, 7).* This indicates that the program was successfully embedded in the existing systems, and the anticipated challenges were not as problematic as initially expected. |
